# Intestinal tuberculosis previously mistreated as Crohn’s disease and complicated with perforation: a case report and literature review

**DOI:** 10.1186/s40064-015-1129-x

**Published:** 2015-07-07

**Authors:** Yu-Feng Wu, Cheng-Maw Ho, Chang-Tsu Yuan, Chiung-Nien Chen

**Affiliations:** Department of Surgery, National Taiwan University Hospital, Taipei, Taiwan; Department of Pathology, National Taiwan University Hospital, Taipei, Taiwan

**Keywords:** Intestinal tuberculosis, Crohn’s disease, Bowel perforation

## Abstract

**Introduction:**

Tuberculosis is known as a notorious mimicker and distinguishing between intestinal tuberculosis and Crohn’s disease is a huge diagnostic challenge.

**Case description:**

Here, we report a case of hollow organ perforation due to intestinal tuberculosis that was previously mistreated as Crohn’s disease. Staged operation with emergency resection of the diseased small bowel and temporary ileostomy was performed for the perforation, followed by 6-month standard treatment for miliary tuberculosis, which was diagnosed on the basis of the presence of acid-fast bacilli in the diseased bowel and positive culture of *Mycobacterium tuberculosis* from sputum, ascites, and stool samples. Ileostomy takedown was performed, and the continuity of the gastrointestinal tract was restored 6 months after the first surgery. The patient recovered well thereafter.

**Conclusion:**

Timely surgical intervention can help establish the finial diagnosis of tuberculosis, rescue the patient from abdominal emergency, and provide a chance for cure.

## Background

Tuberculosis (TB) is known as a notorious mimicker and should be considered before a definite diagnosis has been made, especially in areas where TB is prevalent. Intestinal tuberculosis (ITB) represents the sixth most frequent extra-pulmonary form of tuberculosis, and distinguishing between Crohn’s disease (CD) and ITB is quite a diagnostic challenge. It is estimated that intestinal perforations occur in 1–15 % of all abdominal tuberculosis patients (Lee et al. 2012), frequently requiring surgical intervention. Misdiagnosis followed by inadequate treatment may lead to unsatisfactory and sometimes, catastrophic outcomes, e.g., misapplied immunosuppressants in TB may lead to reactivation of TB or disseminated TB that deteriorates the patient’s condition and prolongs the treatment course. Herein, we report a case of a patient previously misdiagnosed with CD, while his tuberculous intestinal perforation was surgically detected.

## Case description

**Figure 1:**
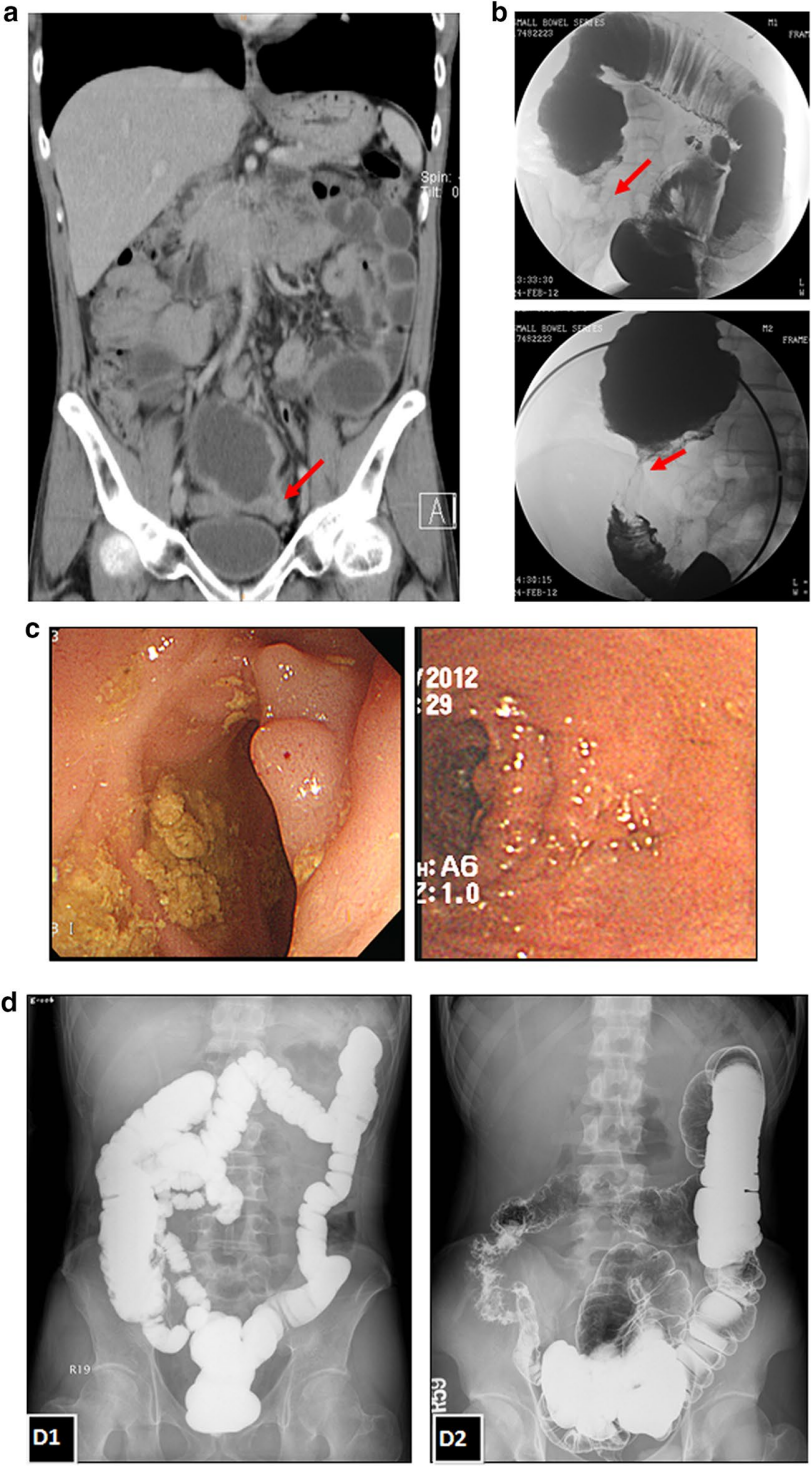
Image studies of the patient with intestinal tuberculosis initially misdiagnosed as Crohn’s disease. **a**–**c** Images for making the initial diagnosis of Crohn’s disease. **a** Coronal view of abdominal computed tomography showed skip lesions with interrupted dilatation of the small bowel, and one stricture point at the terminal ileum with the dilated proximal intestine (*arrow*); **b** Initial barium radiographic study of the intestine showed segmental narrowing of the ileum, which favored post-inflammatory focal stricture (*arrow*); **c** Colonoscopic examination showed polypoid lesion at the terminal ileum. **d** *D1* Barium radiographic study of our patient after completion of anti-tuberculous treatment showing patent intestinal structure; *D2* Barium radiographic study of another 20-year-old male diagnosed with Crohn’s disease showing typical ileocecal valve involvement with cobblestone appearance.

**Figure 2:**
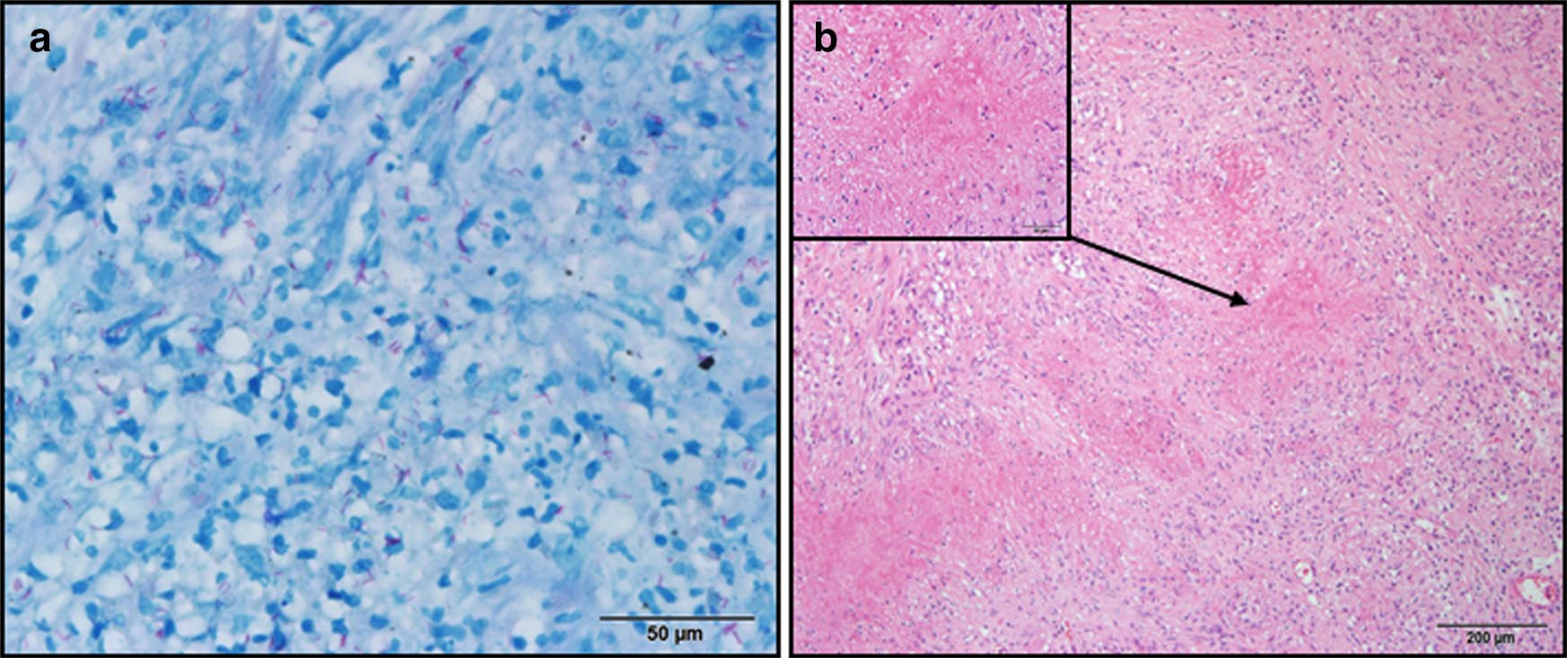
Histopathological examination of the surgical specimen. **a** The presence of acid-fast bacilli in the bowel (acid-fast stain); **b** granulomatous inflammation with caseating necrosis (H&E stain).

A 42-year-old man experienced recurrent abdominal pain and weight loss (15 kg) in 2 years. The diagnosis of CD was made on the basis of abdominal computed tomography (ACT), barium radiographic study of small bowel, and colonoscopic findings. ACT showed skip lesions throughout the small bowel, and suspected healed enteroenteric fistula with multifocal mesenteric adhesion (Figure 1a). Barium radiographic study revealed segmental narrowing of ileum which favored post-inflammatory focal stricture (Figure 1b). Colonoscopic examination showed the presence of polypoid lesions in the terminal ileum (Figure 1c), of which two pieces were biopsied. The pathologic report was chronic inflammatory change without the presence of microorganism. Under the impression of CD, prednisolone and azathioprine were then prescribed but yielded a poor response. He presented to our emergency room with diffuse abdominal pain and intermittent fever 4 months after the initiation of treatment for CD. ACT revealed pneumoperitoneum and hollow organ perforation; the perforated site at the terminal ileum was confirmed by emergent laparotomy. Segmental small bowel resection with end ileostomy was performed for the small bowel perforation. The pathologic report showed granulomatous inflammation with the presence of acid-fast bacilli at the perforated site (Figure 2). Miliary TB was diagnosed with additional positive findings of polymerase chain reaction (PCR) and positive culture for *Mycobacterium tuberculosis* in sputum, stool, and ascites. Tracing back the initial presentation and medical history, no clue of tuberculosis was yielded in early plain films and in clinical presentation. Standard anti-TB treatment was initiated and the patient’s body weight increased, with drastic improvement in abdominal symptoms. Follow-up barium radiographic examination of the intestine (Figure 1d, left) showed no definite residual lesion after completion of his 6-month-course of anti-TB treatment. His body weight returned to his baseline weight of 65 kg, 2 months after ileostomy closure, and there were no abdominal complaints thereafter.

## Discussion and evaluation

TB is a globally prevalent infectious disease, and ITB accounts for 1–3% of all TB cases (Farer et al. 1979). Differentiating between ITB and CD, especially in areas endemic for TB, is quite challenging since both can present as granulomatous inflammation. CD is an inflammatory bowel disease that is characterized by a progressive transmural inflammation with skip lesions throughout the GI tract. Although many presentations of ITB and CD are similar, certain clinical and histological features can be helpful in distinguishing between ITB and CD (Table 1) (Kirsch et al. 2006; Pulimood et al. 1999, 2005). Specifically, the presence of ascites is usually an indication of ITB rather than CD because peritoneal involvement is uncommon in the latter. Serial endoscopic biopsies should be done if feasible. Increasing the number of biopsies and sections examined could increase the diagnostic accuracy. Although the classical histological features of ITB such as caseating granuloma or the presence of acid-fast bacilli are found in less than 30% of the cases (Epstein et al. 2007), combination of endoscopic and histological features could increase the diagnostic rate to around 60% (Bhargava et al. 1992). Culture, which is specific for ITB, often takes several weeks to yield any result. PCR for mycobacterial DNA yields a variable positive rate of 22–75% from biopsy specimens from ITB patients, with a false-positive rate of only 0–5% in CD (Epstein et al. 2007). PCR has a high detection rate in ITB cases without typical granuloma, which is beneficial in histologically atypical cases (Gan et al. 2002). In evaluation of CD, serological antibody markers with perinuclear antineutrophil cytoplasmic antibodies (pANCA) and anti-*Saccharomyces cerevisiae* antibodies (ASCA) may be helpful and have been proposed as a method for diagnosis of inflammatory bowel disease(IBD), including CD. The sensitivity was around 44–60% and the specificity was greater than 90% in distinguishing with control group (Peeters et al. 2001). By reviewing our case, we suggested that more pieces of biopsies during the initial endoscopic examination and further serological tests may decrease the risk of misdiagnosis. Clinical presentation of ITB, the true etiology, may be initially masked by the effect of immunosuppressants used for CD, just like our case. Therefore, ITB should be always kept in mind in this situation and survey, such as culture or PCR for mycobacterial DNA, should be considered in TB endemic areas to prevent further depressing scenario.

**Table 1:** Distinguishing features between intestinal tuberculosis and Crohn’s disease [3–5]

	Intestinal tuberculosis	Crohn’s disease
Clinical presentations		
Age	Any age	20–50
Obstructive symptoms	++	+
Stricture pattern	Short, single	Long, multiple (skip lesions)
Mucosal ulceration	Circumferential	Longitudinal
Fistula	Few	35–50%
Perianal disease	Few	>1/3
Ascites	+	Rare
Histological features		
Caseous necrosis	22–40%	0%
Granulomatous inflammation	78–100%	28–61%
Confluent granuloma	42–60%	0–3%
≧5 granulomas/biopsy	40–45%	0–24%
Large granulomas^a^	51–90%	0–5%
Submucosal granulomas	39–45%	5–12%
Ulcers lined by bands of epithelioid histiocytes	45–61%	0–8%
Disproportionate submucosal inflammation	65–67%	5–10%

^a^Large granuloma: [3]: area > 0.05 mm^2^; [4]: diameter > 400 μm; [5]: diameter > 200 μm.

Although ITB is primarily a medically treatable disease, surgical intervention is indicated in complicated cases, such as obstruction, perforation, or massive hemorrhage. Free perforation occurs in up to 15% of ITB cases, and ITB has been reported to account for 3.9–10% of all cases of small bowel perforation in India, where TB is prevalent; however, lower incidence rate has been reported in developed countries, where TB is uncommon (Jhobta et al. 2006). Three main pathological types of ITB (ulcerative, hypertrophic, and ulcero-hypertrophic) have been reported (Marshall 1993). The majority of ITB perforation is caused by distal obstruction in hypertrophic cases, rather than ulcer perforation. Comparatively, free perforation in CD is a rare complication (1–2%) (Freeman 2002). Whether ITB patients mistreated as CD would exacerbate the clinical course, such as dissemination of TB or increasing the risk of bowel perforation, is unknown. However, therapeutic agents used in CD, such as corticosteroids and anti-tumor necrosis factor monoclonal antibody, were reported to be associated with the increased risk of bowel perforation (ReMine and McIlrath 1980; Eshuis et al. 2012). Immunosuppressants, used in CD, would increase the risk of TB infections, reactivation, or dissemination (Epstein et al. 2007). Whether the bowel perforation risk is higher in disseminated TB than in isolated ITB is not well clarified in the literature.

ITB perforation is difficult to diagnose before surgery. Therefore, specimen from surgical resection at the site of bowel perforation is essential in making the diagnosis. There is no obvious difference in surgical management of free perforation due to ITB or CD. The surgical strategy depends on the extent of the disease and the condition of the patient. If the region involved is limited, the patient’s condition is not toxic, and the residual bowel is relatively healthy, resection of the diseased bowel with primary anastomosis is usually feasible. Otherwise, temporary enterostomy or colostomy with clearance of the sepsis would be preferred. Nevertheless, ITB perforation has a poor prognosis, with mortality greater than 30% (Lee et al. 2012). Regardless of whether surgery is performed, a full course of anti-TB medication should be administered to all patients along with close follow-up (Aston 1997). Among patients with extrapulmonary tuberculosis, a 6- to 9-month regimen (2-month therapy with isoniazid, rifampin, pyrazinamide, and ethambutol followed by 4–7 month therapy with isoniazid and rifampin) is recommended as the initial therapy (American Thoracic Society et al. 2003). The initiation of anti-TB treatment should be timely when TB infection is highly suspected.

## Conclusion

We showed a case of ITB misdiagnosed and mistreated as CD, where the patient presented with intestinal perforation and received emergent surgical intervention. Differentiating between ITB and CD should be given more emphasis, and the similarity between the two conditions should be kept in mind whenever possible, especially in geographic areas endemic for TB or when treating immigrants from these endemic regions, as mistreatment may prolong the ITB course and even lead to fatal complications. The combination of serial biopsies/surgical pathology and endoscopic features is crucial in increasing diagnostic accuracy. Timely surgical intervention in complicated case can not only rescue the patient from abdominal emergencies, but also help establish the true diagnosis, and provide a chance for cure.

## Abbreviations

TB: tuberculosis
ITB: intestinal tuberculosis
CD: Crohn’s disease
ACT: abdominal computed tomography
GI: gastrointestinal
PCR: polymerase chain reaction
IBD: inflammatory bowel disease
pANCA: perinuclear antineutrophil cytoplasmic antibodies
ASCA: anti-*Saccharomyces cerevisiae* antibodies
